# Replacing Plastic with Bamboo: A Review of the Properties and Green Applications of Bamboo-Fiber-Reinforced Polymer Composites

**DOI:** 10.3390/polym15214276

**Published:** 2023-10-31

**Authors:** Dandan Xu, Sheng He, Weiqi Leng, Yuhe Chen, Zaixing Wu

**Affiliations:** 1 China National Bamboo Research Center, Key Laboratory of Bamboo High Efficient Processing of Zhejiang Province, Hangzhou 310012, China; xdandan1995@163.com (D.X.); yuhec@sina.com (Y.C.); jansonwu@126.com (Z.W.); 2 Department of Materials Science and Engineering, Nanjing Forestry University, Nanjing 210037, China

**Keywords:** bamboo fiber, plastic, natural, mechanical

## Abstract

Natural fiber composites are receiving more and more attention because of their greenness and low cost. Among natural fibers, bamboo is characterized by fast growth, a short cultivation period, high strength and good toughness, and is one of the strongest natural fibers in the world. A bamboo-fiber-reinforced polymer composite (BFRPC) has the characteristics of high mechanical strength, low density, degradability, etc. It has the industrial applicability comparable to metal materials, the same strong corrosion resistance as composites such as glass and carbon fibers, and the same immunity to electromagnetic interference and low thermal conductivity as natural materials. Its unidirectional specific strength and unidirectional specific modulus is higher than that of glass fiber, second only to the extremely high price of carbon fiber, which is playing an increasingly important role in the field of composite materials, and can be widely used in the fields of wind power, construction, aviation, automotive, medical care and so on. At present, it has been initially used in packaging, automotive and transportation fields, and is expected to replace petroleum-based plastics in various fields. In addition to their environmental protection and green production, they have excellent physical properties. This paper provides an overview of the mechanical properties of bamboo-fiber-reinforced thermoplastic composites and thermoset composites that have been developed so far, such as tensile strength, flexural properties and impact strength. In addition, the prospects of bamboo-fiber-reinforced thermoplastic composites for automotive, packaging and agricultural applications are presented.

## 1. Introduction

Plastic usage is widespread in the packaging, aerospace, marine, and automobile industries because of their lightweight, low manufacturing costs, corrosion resistance and exceptional specific mechanical properties [1]. However, most plastics are not naturally degradable, and these non-renewable resources have increasingly expanded from land to sea, posing a huge threat to the environment and human health, and the high costs of petroleum-based plastics restrict their use in other applications [2]. With the rapid development of technology and the increasing degree of industrial mechanization, the shortage of fuel and energy has become an important problem that needs to be solved urgently [3]. The storage capacity of non-renewable resources such as oil, coal and natural gas is unpromising, which will inevitably lead to the depletion of functional materials synthesized from non-renewable chemical resources [4]. Therefore, eco-friendly alternative materials for plastic are needed to solve these problems.

Fiber composites can be classified as synthetic fiber composites and natural fiber composites. Synthetic fiber composites such as carbon fiber, basalt fiber, and glass fiber are widely used in aerospace, construction, marine and other fields due to their excellent performance. Carbon fiber has extremely high mechanical properties, excellent fatigue resistance, corrosion resistance, high temperature resistance and creep resistance, a good electromagnetic shielding effect, and can be applied to aircraft. Basalt fiber has good acid and corrosion resistance, good mechanical properties, and abundant sources. Glass fiber is light in quality, has good tensile strength, and has excellent performance in electric insulation, high temperature resistance, flame retardance and heat insulation [5,6,7]. But synthetic fiber composites are costly and non-renewable, and their widespread use has had a significant negative impact on the ecology.

Using natural-fiber-based composites is a feasible alternative. Since natural fibers are biodegradable, readily available, eco-friendly, resourceful, low-cost, and increase productivity, offer high specific strength and stiffness, etc. [8,9]. Natural fiber-reinforced composites have become a hot research topic due to their environmental friendliness and high performance [10]. And natural fiber composites have made a difference in packaging, aerospace, automotive, etc. [11], which is has important implications for the green economy and low-carbon development [12,13,14,15].

## 2. Natural Fibers

**Figure 1:**
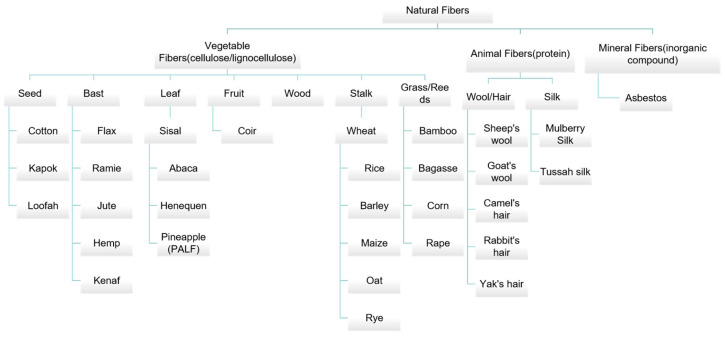
The classification of natural fibers.

Natural fibers are fibers obtained directly from plants or animals. It can be categorized into vegetable fibers, animal fibers, and minerals fiber based on their origins [16]. The detailed classification of natural fibers is shown in Figure 1. The main component of plant fiber is cellulose, and animal fiber consists mainly of protein.

Natural fiber composites are renewable resources and therefore available easily, and require one-third of the energy needed to produce glass fibers, which can reduce production costs. Plant fibers absorb as much carbon dioxide during growth as they release when they decompose, which is in line with decarbonization development requirements. In the processing of natural fibers, the process is harmless and there is virtually no wear and tear on the tools and no skin irritation caused by the glass fiber process. Natural fibers have a higher specific strength and stiffness than glass and a lower density, making them lighter in weight. The mechanical properties of natural fiber composites are 25% to 30% stronger than glass fibers of the same weight. Composites made from natural fibers help to reduce the weight of components, thereby reducing total energy consumption [17,18,19]. Table 1 summarizes the mechanical properties of natural-fiber-reinforced composites.

Due to the special properties of natural fibers, their applications cover almost all sectors of the economy, largely contributing to green consumption. Plant fibers play an important role in textile, pulp and paper industry and biodegradable composite applications [29].

In addition to these traditional applications, in recent years, with the increased awareness of environmental protection and the need for sustainable development, there have been an increasing number of natural-fiber-based composites used in packaging, automotive, aerospace and other applications [18,30,31].

Wood–plastic composites (WPC) have been used in many fields because of their environmental friendliness [32]. They have good mechanical properties and are easy to process, and the material itself can be recycled, making it a new green product and an eco-friendly composite material. Wood–plastic products are now mainly used in the furniture decoration, industrial structure, construction and packaging industries. For example, in outdoor landscapes, wood–plastic composites are commonly used in the construction of guardrails, pavers, and landscape vignettes [33,34,35].

Natural fiber composites are also playing an increasingly important role in the automotive industry. For 2019, Porsche manufactured the racing car’s sector doors and rear wing from natural fiber composites. In 2020, the Porsche 718 Cayman GT4 Clubsport MR made its debut in the Nürburgring 24 h race car with a complete body kit constructed from natural fiber composites. Ishak et al. reduced the weight of the front hood of the car by using natural fiber metal laminates. This also helps in reducing the engine gasses emissions and helps in reducing the impacts associated with global warming [36].

Natural fiber composites are also used extensively in packaging [37,38,39]. In 2018, researchers at Georgia Tech extracted chitin from crab shells and cellulose from trees. The researchers suspended chitin nanofibers and cellulose in water and through multiple spraying to obtain a transparent film, where the film after drying was transparent soft and tough and can be completely degraded. Preparation of transparent film may be used to replace plastic for food packaging, and this research is of great significance in solving the problem of white pollution caused by plastic. In another study, Sanyang et al. successfully prepared a flexible bilayer film by combining sugar palm starch (SPS) and polylactic acid (PLA). The bilayer film is biodegradable, and as a green biopolymer, it can be used to develop bio-based packaging materials for food packaging, solving the problem of pollution caused by traditional plastics [40].

As one kind of biomass resource, bamboo is characterized by rapid growth, abundant resources, one-time planting for perpetual utilization, green and low-carbon, and is an excellent renewable resource. The investigation and utilization of bamboo is conducive to the sustainable development of society.

## 3. Bamboo Fiber

**Figure 2:**
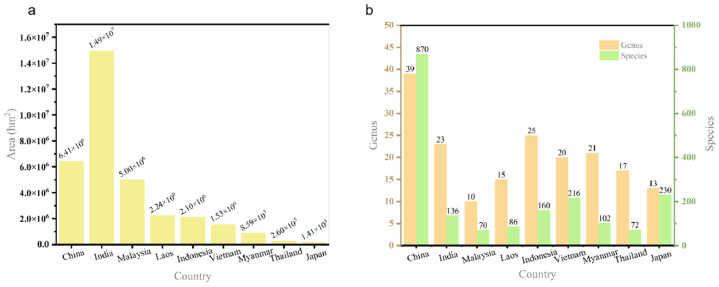
Area of bamboo forests (**a**), number of bamboo genera and species (**b**) in the major bamboo-producing countries of the Asia-Pacific Bamboo Region.

According to the latest statistics, there are 127 genus and more than 1680 species of bamboo worldwide, which are mainly distributed in tropical and subtropical regions, and a few of them are distributed in the temperate zone and frigid zone [41]. The geographical distribution of bamboo in the world can be divided into three major bamboo regions, namely the Asia-Pacific Bamboo Region, the Americas Bamboo Region, and the Africa Bamboo Region. The Asia-Pacific Bamboo Region is the largest bamboo region in the world, with about 50 genera and 900 species of bamboo. The main bamboo-producing countries include China, India, Thailand, Myanmar, Bangladesh, Cambodia, Vietnam, Japan, Indonesia, Malaysia, the Philippines, South Korea and Sri Lanka. China is the world’s leading bamboo-producing country, with a large variety of bamboo species, a wide area and high economic value. There are 39 genera and over 870 species of bamboo plants in China, and the existing bamboo forests cover an area of 6.4116 million hm^2^. Figure 2 compares the area of bamboo forests and the number of bamboo genera and species in the major bamboo-producing countries of the Asia-Pacific Bamboo Region.

Currently, the world’s forest resources and forest areas are decreasing dramatically, but the area of bamboo forests is still growing at a rate of 3% per year. At present, the global bamboo forest area has reached 32 million hectares, accounting for about 1% of the forest area, and the annual bamboo production can reach 40 million tons.

**Figure 3:**
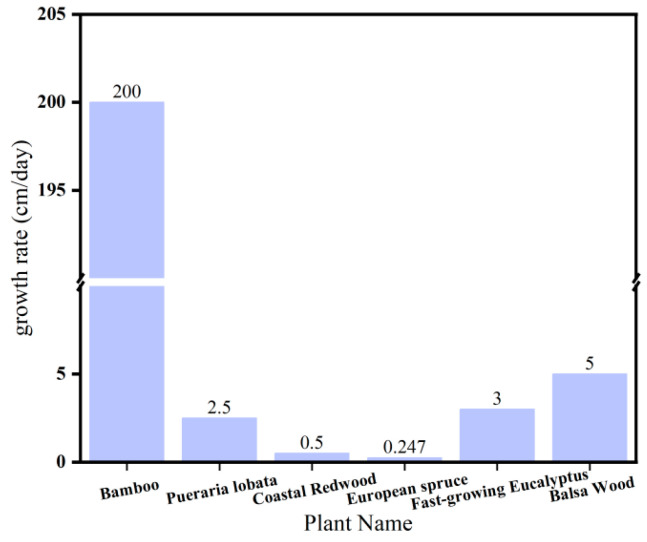
The growth rate of bamboo and other fast-growing plants.

Bamboo grows vigorously. While a 20 m tall tree may take up to 60 years to grow, a 20 m tall bamboo only takes about 60 days. Bamboo can grow up to two meters a day, much faster than many trees known for their fast growth [42,43,44]. Figure 3 shows the growth rate of bamboo and other fast-growing plants.

## 4. Advantages of Bamboo Fiber

Bamboo fiber is a kind of natural fiber, and bamboo is rich in resources, with green, low-carbon, recyclable characteristics, thus using bamboo fiber to manufacture bamboo fiber-based composite materials to replace plastic is conducive to the promotion of a green economy and ecological civilization [45,46]. With global warming and increasing environmental threats, forest resources are becoming more and more precious. Bamboo’s rapid growth cycle and abundant resources make the “bamboo instead of plastic” industry promising.

Bamboo fiber has high strength, good elasticity and low density, and its fibers always keep axial growth. The specific strength of bamboo fiber is 2–3 times that of steel, with high tensile and compressive strength [43,44,47]. Many plant fibers have been reported for composite reinforcement, such as sisal, flax, ramie, cotton fiber, bamboo fiber, and sugarcane fiber. Among them, bamboo fiber has good development potential due to its high yield, high specific strength and modulus, and light weight and low price [6]. The mechanical strength of bamboo fiber is relatively greater among plant fibers (Table 2).

Bamboo can minimize greenhouse gas emissions in a number of ways. Bamboo grows fast and has a high capacity for carbon sequestration [48]. Bamboo products can store carbon for a long time and maintain a low or even zero carbon impact throughout the product life cycle. The same area of bamboo forest can release 35% more oxygen than a forest, which means that the bamboo industry is not only a low-carbon industry, but also a carbon-negative industry. Bamboo can reduce greenhouse gas emissions by replacing energy-intensive and emission-intensive plastics, concrete and steel.

**Table 2:** Mechanical properties of different natural fibers. Sources: Yan, Chouw and Jayaraman [49]; Onuaguluchi and Banthia [50]; Ramakrishnan et al. [51]; Sanjay, Arpitha, and Yogesha [52]; Ku et al. [53]; Vaisanen et al. [54]; Sagar et al. [55].

Fiber Type	Density/(g/cm^3^)	Tensile Strength/MPa	Elastic Modulus/GPa	Elongation at Break/%
Bamboo fiber	0.6–1.1	140–800	11–35.9	1.3–7
Jute	1.3–1.5	187–800	3–55	1.3–3
Flax	1.2–1.5	343–2000	23.9–103	2.7–3.2
Sisal Hemp	0.7–1.5	350–840	9–38	2–14
Ramie	1–1.5	220–1000	23–128	1.2–4
Bagasse	0.5–1.2	20–350	0.5–27	0.9–5.8
Coir	1.2–1.5	95–593	2.8–6	15–51.4
E-glass	2.5–2.59	2000–3500	70–80	2–3

## 5. Extraction of Bamboo Fiber

**Figure 4:**
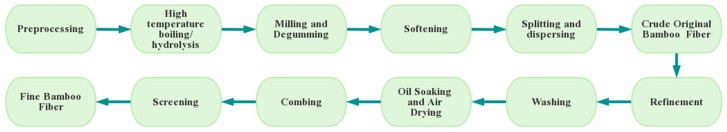
Natural bamboo fiber production line.

Bamboo fiber can be categorized into regenerated bamboo fiber and natural bamboo fiber. Regenerated bamboo fiber is also known as bamboo pulp fiber (BPF) or viscose fiber, and is the spinnable fiber made by adding chemicals to bamboo pulp and reprocessing it. Natural bamboo fiber, also known as original bamboo fiber, is extracted by removing lignin, pectin, polysaccharides and other components of the bamboo material through physical and mechanical methods [56,57]. Original bamboo fiber and BPF have different process routes, and the process route of original bamboo fiber is shown in Figure 4.

**Figure 5:**
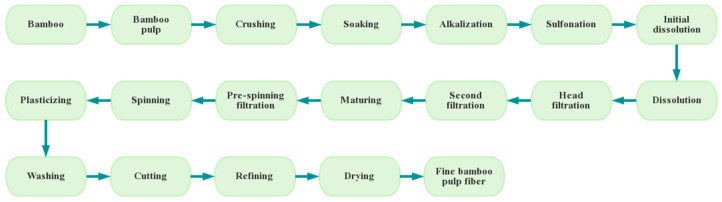
The route of bamboo pulp fiber production process.

The production process of BPF is distinct from original bamboo fiber. Original bamboo fiber is processed by mechanical and physical methods on bamboo material without adding any chemicals, and the processing is environmentally friendly, non-polluting and has a low production cost. BPF requires a large number of chemicals to pre-treat the bamboo material to dissolve lignin, pectin and other substances, and the processing will generate wastewater, which is hazardous to the ecological environment. In addition, the use of chemicals raises the production cost, and the production procedure is complicated and long-term. The production process of BPF is presented in Figure 5.

Bamboo fiber extraction methods include physico-mechanical, biological and chemical methods [58]. Mechanical methods include hammering or crushing method, mechanical disintegration method, and steam explosion method [59]. However, the fiber length and fineness produced by these methods are not uniform, and the fibers are short and brittle, with poor toughness, making it difficult to fulfill the requirements of textile fibers. The chemical method extracts bamboo fiber by dissolving the gums, lignin and other substances in the bamboo material with chemicals. The fibers produced by this method show low tensile strength, and the residual chemicals are harmful to human health. The biological method is to degum bamboo material and bamboo fiber by biological enzyme or hemicellulase. However, the biological activity of the enzyme is affected by conditions such as temperature, acidity and alkalinity, and the processing time is long and the efficiency of fiber production is low. The biological method has not yet achieved breakthrough research achievements, so it is not suitable for application in large-scale production [57,60,61,62].

Xinzhou et al. pretreated fresh bamboo poles by saturated steam at 180 °C and then mechanically rolled and twisted in a multi-roll mill to obtain a bamboo fiber bundle (BFB). This method caused less damage to the microstructure of the bamboo fiber cell walls, and the isolated BFBs had lower hygroscopicity and improved cell walls’ mechanical properties. It has significant tensile property advantages and can effectively improve the bending strength of laminated veneer wood [63]. Many studies prepared bamboo fibers by combining mechanical and chemical methods to improve the mechanical properties of bamboo fibers. For instance, Hsuan et al. adopted a mechanical method to extract bamboo fibers and soaked the obtained bamboo fibers in sodium hydroxide solutions with concentrations of 0.5 wt%, 2.5 wt% and 5 wt% for 2 h at room temperature to remove impurities such as lignin and hemicellulose. The obtained bamboo fiber interface has enhanced shear strength. As a reinforcement for the bamboo-fiber-reinforced epoxy resin (BF/EP) composites, it also improved the tensile strength of the composites. The obtained bamboo fiber has higher interfacial shear strength. As a reinforcement, it also improved the tensile strength of the composites [64].

## 6. Category of Bamboo Fiber-Reinforced Polymer Composites

BFRPC is an environmentally friendly composite material prepared by molding bamboo fibers with a thermosetting or thermoplastic resin matrix. BFRPC matrix types are generally categorized into thermoplastic polymers (polyethylene, polypropylene, polyvinyl chloride, etc.) and thermoset polymers (polyurethane, epoxy, phenolic, unsaturated polyester, etc.). BFRPC can be classified into bamboo-fiber-reinforced thermoplastic composites and bamboo-fiber-reinforced thermosetting polymer composites according to the type of matrix. Bamboo-fiber-reinforced thermoplastic composites have high specific strength, easy processing, corrosion resistance and good fatigue resistance. Bamboo-fiber-reinforced thermosetting polymer composites are characterized by strong hardness, high rigidity, high temperature resistance, and not easy to deform.

The physical properties of BFRPC are related to the density of bamboo fibers and bamboo fiber bundles, and the mechanical properties such as flexural strength and tensile modulus of BFRPC with high fiber density are higher. The species of bamboo and its age also have some influence on the physical and mechanical properties of BFRPC [65,66,67]. In the practical production of BFRPC, the influence of these factors should also be taken into account so as to select the appropriate bamboo material.

The processing and preparation technology of BFRPC mainly includes four steps of raw material treatment, mixing, granulation and molding [56,68]. The molding process mainly includes extrusion molding, hot press molding and compression molding [69].

## 7. Properties of Bamboo Fiber-Reinforced Polymer Composites

Compared with other wood fibers, the cellulose content of bamboo fiber is lower, and the content of lignin and hemicellulose is much higher than that of other wood fibers such as ramie and flax [70]. Bamboo fiber has a larger aspect ratio and specific surface area, its fibers are strongly entangled and interwoven, and the bonding strength between the fibers is greater, so the fiber strength is higher than most wood fibers. Bamboo fiber as a reinforcing material can effectively improve the tensile strength and impact strength of polymer composites [71].

Bamboo fiber absorbs sound better than carbon and glass fibers and provides better thermal insulation than carbon fiber. BFRPC has industrial applicability comparable to metal materials and its corrosion resistance is as strong as that of glass and carbon fiber composites. It is as immune to electromagnetic interference as natural materials and has a low thermal conductivity. Its unidirectional-specific strength and unidirectional-specific modulus is higher than that of glass fiber, second only to the extremely high price of carbon fiber. And its manufacturing cost is much lower than the popular stainless steel and aluminum alloys on the market, which has great development potential [72,73,74]. Moreover, because of its light weight and high strength, environmental friendliness and low energy consumption, it can replace glass fiber and polymer fiber, which is a green and sustainable fiber-reinforced material that can be used in various fields such as construction, leading to the reduction of carbon emission. Bamboo fiber as a reinforcing material can effectively improve the tensile and impact strength of polymer composites. BFRPC is clearly superior to glass fiber composites in terms of density, cost, energy consumption and environmental friendliness.

### 7.1. Interfacial Modification of Bamboo Fiber-Reinforced Polymer Composite

BF is hydrophilic and the matrix is hydrophobic, and the hydroxyl group cannot effectively react with the polymer matrix [75], which results in interfacial incompatibility, and further leads to uneven dispersion of fibers in matrix, weak interfacial bonding of composites [76,77], and poor interfacial adhesion [78]. Ultimately, the composite material presents undesirable consequences such as debonding. This will adversely affect the mechanical properties of the composites [79,80]. In order to improve the mechanical properties of the composites, the interfacial strength between the bamboo fibers and the matrix can be enhanced by mechanical, chemical or a combination of both techniques [81]. Mechanical interface modification methods mainly include particle filling [82,83,84,85,86,87,88], plasma treatment [89,90], and heat treatment [91,92]. Chemical methods such as adding coupling agent [93,94,95,96], graft copolymerization [20,97,98], alkali and acid treatment [99,100,101,102] and so on.

Wei et al. [103] modified the surface of bamboo powder by silane KH550, and prepared high-performance bamboo powder/silane KH550-HDPE composite by pretreatment/granulation/molding process. Characterization of the material by diffractometer, spectrometer, microscope and calorimeter showed that after 6 wt% KH550 treatment, the crystallinity of the bamboo material was increased by 1.11%, and the melting temperature and enthalpy of the composite material were increased by 2.37 °C and 5.27 J/g, which was consistent with the improvement of the interfacial morphology. The thickness was increased from 3 mm to 9 mm and the mechanical properties of the composites were generally improved. In 2021, Wei et al. [104]. further successfully attached biomimetic polydopamine (PDA) coatings to bamboo flour (BF) using different oxidizing agents, such as O_2_, CuSO_4_, NaIO_4_, (NH_4_)_2_-S_2_O_8_, and FeCl_3_, which enhanced the surface free energy and the peak temperature of thermal degradation of the BF, and improved the water-wettability, surface roughness, and specific surface area of the BF. This resulted in enhanced interaction between BF and HDPE, which strengthened the mechanical properties of the composites.

In another study, Huang et al. [105] modified bamboo particles with varying degrees of acetylation by immersing them in dimethylformamide solutions containing acetic anhydride (acetylated) or butyric anhydride (butyrylated) and potassium acetate. The interfacial bonding between the modified bamboo particles and the polymer matrix was significantly improved. The bamboo particles/high density polyethylene (BP/HDPE) composites were then produced by compression molding. The mechanical properties and chemical composition of the composites were tested and analyzed, and the results showed that esterification had a significant effect on the mechanical and interfacial properties of the composites.

Mechanical and chemical combination methods are also widely used for interfacial modification of bamboo–plastic composites. For instance, Li et al. [106] obtained a lightweight and high-strength structural material made from natural bamboo by delignification and mechanical pressing. Hollow bamboo was first softened by high-pressure steam, then large blocks of bamboo were soaked in a boiling water solution of sodium hydroxide and sodium sulfite for 12 h to partially remove lignin and hemicellulose, and then the bamboo was hot-pressed to densification. The tensile strength and tensile modulus of the resulting high-density bamboo reached a record high of 1 GPa and 75 GPa, due to the low density of bamboo (1.35 g/cm^3^), and the specific strength of the bamboo reached 777 MPa cm^3^/g, which is much higher than that of other natural materials, stainless steel, high-alloy steel and titanium alloys.

### 7.2. Properties of Bamboo-Fiber-Reinforced Thermoplastic Composites

In recent years, the research on bamboo-fiber-reinforced thermoplastic composites has been increasing, and the mechanical properties of bamboo-fiber-reinforced thermoplastic composites are summarized in Table 3. Abhilash et al. [107] fabricated bamboo-fiber-reinforced polypropylene (PP) composites using homopolymer and copolymer PP as the matrix and 10% polyolefin elastomer (POE) as the impact modifier. Investigated the effect of elastomer on the mechanical properties of the composites. The addition of 10% elastomer to homopolymerized PP composites with 30% bamboo content resulted in a 60% increase in elongation at break and a 29% increase in unnotched impact strength as compared to composites without added elastomer. A copolymer matrix with 10% elastomer gives 37% higher strength and modulus than a homopolymer (10% POE) 30% bamboo composite even at 50% bamboo content. The properties of bamboo fiber composites can be adjusted for different applications by varying the ratio of reinforcement and elastomer. Wang et al. alkali-treated bamboo fibers and then immersed them in graphene oxide (GO) suspension, stirred and dried to obtain graphene oxide grafted bamboo fibers with different weight ratios. Finally, the graphene-oxide-modified bamboo-fiber-reinforced polypropylene composites were prepared by a microinjection molding technique. Compared with the untreated bamboo fiber (UBF)/PP composites, the tensile strength of 0.1 GO-ABF/PP composites increased from 31.9 MPa to 35.9 MPa (12.6% increase), and the flexural strength increased from 42.9 MPa to 53.1 MPa (23.7% increase) [96]. Bamboo original fiber (BOF) and bamboo viscose fiber (BVF) were used to reinforce PP. Compared with flax fiber composites, bamboo-fiber-reinforced composites are more environmentally friendly. The addition of maleic-anhydride-grafted polypropylene improved the mechanical and environmental properties of bamboo-fiber-reinforced composites [20].

The bamboo pulp fiber/high density polyethylene composites were modified by nano CaCO_3_ mixing and impregnation techniques, and the effects of two different modification methods on the mechanical properties of the composites were investigated. The composites were produced by three different processes: hot pressing, extrusion and injection molding. The results showed that the impregnation modification had a greater effect on the rheological properties of the composites than the nano-CaCO_3_ blend modification, and had the greatest effect on the properties of the extruded composites [108]. BPF or white mud (WM) was added to HDPE-based bamboo plastic composites (BPCs). When BPF was added to BPCs, the flexural, tensile and impact properties of the composites were improved. The addition of WM improved the flexural properties of BPCs but decreased the tensile and impact strengths [109].

Talc-filled EPDM (ethylene–propylene–diene monomer) toughened polypropylene (PP) is widely used in the automotive industry for manufacturing automotive interior and exterior components. The addition of bamboo fibers and maleic anhydride at different levels to recycled PP/EPDM/talc composites was found to significantly increase the tensile and flexural strengths, and the addition of maleic anhydride positively affected the tensile strength, flexural strength and fatigue life of the composites [110]. In another study, sugar-sourced precipitated calcium carbonate (PCC) and bamboo fibers were used as fillers in the matrix of recycled polypropylene and polyethylene (R-PP/PE) resins. With the PCC content ranging from 15% to 30%, the tensile and flexural modulus of the bamboo–polymer composites increased with the increase in PCC content. At high PCC content, the modulus values of PCC–bamboo–polymer composites are 3–4 times higher than those of PCC–polymer composites [111].

PLA is a biocompatible and biodegradable polyester, and BF-PLA composites have been extensively studied by researchers to compete with petroleum-based polymers. Dopamine (DA) can be used as a coupling agent to improve the interfacial adhesion of BF/PLA composites, which in turn improves the mechanical properties of the composites. Alkali treatment also had a positive effect on the mechanical properties of the composites. Co-treatment of the composites with 4 wt% NaOH solution and 1 wt% concentration of DA improved the thermal and mechanical properties of the composites compared to unmodified BF/PLA composites. The flexural strength, tensile strength and impact strength were increased by 16.1%, 34.4% and 3.7%, respectively [112]. In another study, the surface modification of high temperature ultrafine bamboo charcoal fiber (UFBC) was carried out by Qian et al. [113] with different concentrations of HNO_3_ and NaOH solution. The mechanical properties of the modified PLA/UFBC composites were also tested, and it was found that the composites had the highest mechanical properties with maximum tensile strength, elongation at break, and impact strength (65.36 MPa, 15.36%, and 25.69 kJ/m^2^) when the concentration of NaOH was 15 wt%, and the concentrations of HNO_3_ were 39 and 54 wt%. At 30% bamboo charcoal fiber content, the maximum values of tensile strength, tensile modulus and impact strength of BC/PLA composites reached 14.03 MPa, 557.74 MPa and 20.50 J/m^2^, respectively, and the impact strength was increased by 160% compared with that of pure PLA [114].

**Table 3:** Mechanical properties of bamboo-fiber-reinforced thermoplastic polymer.

Thermoplastic Matrix	Fiber Addition (wt%)	Modifiers	Tensile Strength (MPa)	Flexural Strength (MPa)	Impact Strength (kJ/m^2^)	Elongation at Break (%)	Reference
PP	30–50	Polyolefin Elastomer	42.89	54.26	12	6.91	[107]
HDPE	30	Nano CaCO_3_	45.09	59.17	40.95	—	[108]
PP/EPDM/talc	20–40	Maleic anhydride	26.14	39.1	7.47	6.96	[110]
PP	42	Alkaline treatment	35.9	53.1	—	—	[96]
PP	10–30	Alkaline treatment and Maleic anhydride	30.85	91.03	—	—	[115]
HDPE	20–40	Maleic anhydride	—	56.47	7.07	7.44	[109]
R-PP/PE	40	Silane treatment	—	34.58	4.33	—	[111]
PP	40	Maleic anhydride	61.85	66.25	70.75	—	[20]
PLA	40	Dopamine hydrochloride and alkaline treatment	39.51	64.25	8.43	—	[112]
PLA	2.5	Alkaline treatment	65.36	—	16.05	5.36	[113]
PLA	0–40	Untreated	14	—	21	2.5	[114]

### 7.3. Properties of Bamboo Fiber-Reinforced Thermosetting Polymer Composites

The mechanical properties of various bamboo-fiber-reinforced thermoset composites are exhibited in Table 4. Martijanti et al. [115] fabricated bamboo-particles-reinforced polyester composites, the tensile and flexural strength of the composites increased with the increase in the volume fraction and size of bamboo particles. When the particle size of bamboo particles is 250 mesh and the volume fraction is 30%, the bending strength and tensile strength of the composites reach the maximum of 91.03 MPa and 30.85 MPa. It is promising to be used as a material for particle board products. In another study, bamboo particles with 0–5% content were incorporated into unsaturated polyester along with a woven/nonwoven kenaf fiber mat. The addition of a small amount of nanoparticles (up to 3%) enhanced the interfacial bonding between the matrix and fibers and improved the tensile strength, flexural strength, impact strength, fatigue life and thermal stability of the composites. However, when the concentration of bamboo particles exceeded 3%, the interfacial adhesion between the fiber and matrix became poor due to the aggregation of nanoparticles, resulting in a decrease in the mechanical properties of the samples [116]. In 2017, Kumari et al. [117] modified bamboo fiber/polyester composites with euphorbia coagulum (EC) as a binder using compression molding technique, and the mechanical properties of the composites were improved by alkali treatment to change the bamboo fibers from hydrophilic to hydrophobic. The best mechanical and thermal properties of the composites with low water absorption were obtained when the bamboo fiber content was 40% and EC content was 30%.

BF/EP composites were prepared by alkali treatment of bamboo fibers with 42% addition. The alkali-treated BF/EP composites obtained better tensile strength than the untreated BF/EP composites. The tensile strength and Young’s modulus of the composites increased with the decrease in the diameter of bamboo fibers [118]. Khan et al. improved the interfacial adhesion of BF/EP composites by treating bamboo fibers with 6% NaOH, and the tensile strength of the composites reached a maximum of 234.6 MPa. The length of bamboo fibers also had a significant effect on the mechanical properties of BF/EP composites, and the toughness at fracture (K_IC_) values of the BF/EP composites with 25 mm bamboo fibers were higher than those with 10 mm and 20 mm. BF/EP composites were developed by a hand-layup method with reinforcement of bamboo fibers of different sizes, such as bamboo chips, whole bamboo, bamboo nodes, and bamboo strips. The impact, tensile and flexural strengths of the composites were enhanced with the increase in fiber content. However, excessive fiber rather affects the interfacial bonding of the composites, which in turn reduces the mechanical properties of the composites [119]. Kumar et al. fabricated bamboo-fiber-reinforced epoxy (BFRE) composites with a nanoclay content ranging from 0 to 10 wt% and the best mechanical properties of the composites were obtained with a nanoclay content of 3 wt%. As compared to pure BFRE, the tensile strength and modulus increased from 80 MPa and 6400 MPa to 112 MPa and 6825 MPa, the flexural strength and flexural modulus increased from 105 MPa and 11,200 MPa to 134 MPa and 14,300 MPa. The tensile and flexural strengths increased by 40% and 27%, respectively [120]. The hybrid epoxy composites of glass/bamboo fiber reinforced with inorganic fillers (TiO_2_ and ZrO_2_) were prepared by a hand stacking technique. The addition of glass to the bamboo fiber composites enhanced the properties of the resulting hybrid composites. The position of the bamboo layer in the bamboo/glass hybrid laminates affected the tensile and flexural strength of the composites. The addition of ZrO_2_ gave the composites better strength and abrasion resistance compared to TiO_2_-filled composites. And the best mechanical properties of hybrid GBBG laminated composites were obtained when the ZrO_2_ content was 9% [121].

Resin content has a great influence on the properties of outdoor bamboo-fiber-reinforced composites (OBFRC). The bamboo fibers of OBFRC were obtained by mechanical separation, and the matrix was phenol formaldehyde (PF) resin at concentrations of 10, 15, 20 and 25 wt%. The water resistance, shear strength and compressive strength of OBFRC at different matrix concentrations were tested, and it was found that the highest mechanical properties of OBFRC were obtained at 20% PF concentration. The mechanical properties of OBFRC at all PF concentrations (10–25%) were better than those of bamboo-based composites [122]. In another work, high performance palm oil (PO)-based thermoset resins were prepared by copolymerizing palm oil fatty acid ethyl acrylamide (POFA-EA) with natural phenolic (NP) cross-linkers. And highly bio-based composites were developed using these thermosetting resins as a matrix and bamboo fibers as the reinforcement. The prepared biocomposites have comparable mechanical properties and thermal stability to petroleum-based biocomposites and are degradable and environmentally compatible [123].

**Table 4:** Mechanical properties of bamboo-fiber-reinforced thermosetting polymer.

Thermosetting Matrix	Fiber Addition (wt%)	Modifiers	Tensile Strength (MPa)	Flexural Strength (MPa)	Impact Strength (kJ/m^2^)	Elongation at Break (%)	Reference
Polyester	10–30	Alkaline treatment	27.4	49.71	—	—	[115]
Polyester	3	Untreated	55.68	75.6	7.43	8.97	[116]
Polyester	25–40	Alkaline treatment	16.29	39.87	4.7	—	[117]
EP	42	Alkaline treatment	222.71	182.29	—	2.23	[118]
EP	20	Alkaline treatment	234.6	—	—	—	[124]
EP	30	Untreated	18.07	—	3.0	—	[119]
EP	68	Untreated	113	131	60	—	[120]

## 8. Applications of Bamboo-Fiber Reinforced Polymer Composites

Plastic waste pollution has become one of the most serious environmental pollution crises on Earth. In recent years, more and more researchers are developing and producing bamboo products to replace plastic products, such as bamboo winding composite pipes, disposable bamboo tableware, car interiors and so on. So that bamboo products instead of plastic meet the needs of people, but also accord with the requirements of green environmental protection, they promote the “bamboo instead of plastic” industry development.

### 8.1. Application of Bamboo-Fiber-Reinforced Polymer Composites in Automotive

Parvez et al. extracted bamboo and rattan fibers by a tanning process and fabricated bamboo fiber composites, rattan fiber composites, and rattan–bamboo fiber composites by a vacuum bagging technique. The mechanical strength of rattan–bamboo fiber composites was found to be higher than that of other composites through testing and comparison, and its flexural strength and impact strength were the largest, which were 57.66 MPa and 44.49 kJ/m^2^, respectively. At the same time, the mechanical properties of the fabricated composites were compared with those of the ABS plastic materials, and the results showed that the mechanical properties of rattan–bamboo fiber composites were better, and they were expected to be applied in the field of automobiles [125].

The tensile strength of the composites was improved by adding 5, 10 wt% of portunus filler to the bamboo-fiber-reinforced titanium composites. The bamboo-fiber-reinforced titanium composites were prepared by a hand lay-up process. The experimental results show that the average tensile strength of bamboo fiber composites filled with 10 wt% portunus filler is 63.6965 MPa, which is about twice as much as that of unfilled bamboo fiber composites [126]. The fabricated composites can be used in automobile hoods and engine covers.

Carbon fiber (CF), bamboo fiber (BF) and polypropylene (PP) fiber hybrid fiber mats were prepared by wet-laid (WL) technique and bamboo fiber-carbon fiber-polypropylene composites (BF-CF-PP) were prepared by compression molding. The mechanical properties of the composites were investigated for different fiber–resin weight percentage formulations. The effects of fiber length, surface treatment, fiber content and consolidation pressure on the mechanical properties of the composites were evaluated. The results indicate that BF-CF-PP (8/32/60) has higher mechanical strength and the mechanical properties of the composites can be modulated by varying the stoichiometric ratio of bamboo and carbon fibers to provide higher performance and environmentally friendly benefits of automotive components [127].

BFRPC is characterized by low density, high modulus and high strength. And compared to traditional composites, BFRPC is lighter in mass, consumes less energy to produce, and emits less carbon, which makes it ideal for automotive components. Dawit et al. developed bamboo and sisal-fiber-reinforced polyester hybrid composites (BSFRHC) using a hand-lay-up technique at a ratio of 3:1 of bamboo and sisal fibers. The properties of BSFRHC with three different fiber orientations were experimentally determined and impact analysis of automotive interior door panels made of BSFRHC was carried out using ANSYS software. The study found that the unidirectional 0-fiber BSFRHC has the potential to replace traditional plastic parts in automotive interior applications [128].

### 8.2. Application of Bamboo Fiber Reinforced Polymer Composites in Packaging

A combination of steam explosion technology and mechanical refining is used to separate bamboo materials, and then the bamboo fibers are mixed with tapioca starch to prepare new bamboo fiber tableware. The prepared product has the characteristics of high strength, water resistance, oil resistance, temperature resistance, dissolution resistance, etc., and can be quickly and harmlessly degraded, which is an ideal substitute for traditional non-degradable plastics in food packaging applications [48].

Luan et al. [129] developed an efficient, automated, mechanized and industrialized process including bamboo stick pretreatment, hydrothermal treatment and bamboo stick drilling to produce bamboo drinking straws. The production process is chemical-free, which not only preserves the natural structure and biodegradability of bamboo, but also has good mechanical properties with a maximum compressive strength of 71.65 MPa and flexural strength of 123.90 MPa. The bamboo straws produced have good wetting stability, high recyclability and low production cost, which is of great significance to alleviate the “white pollution” caused by plastic straws.

Bamboo fiber dinnerware (BFD) is lightweight, high strength, and its specific strength and specific modulus are 4.50 times and 3.09 times of PLA dinnerware, respectively. The glass transition temperature of BFD dinnerware is 63.2 °C, and the activation energy is 70.76 kJ/mol, while that of PLA is 54.7 °C and 132.53 kJ/mol, which means that BFD has better thermal stability, which makes it a good environmentally friendly disposable tableware [130].

A multilayer coating was formed on the surface of bamboo fibers by alkali pretreatment, synergistic treatment with polydopamine and silane coupling agent. And the high-strength bamboo-fiber-reinforced composites were made from polylactic acid and synergistically treated bamboo fibers using a hot pressing technique. The tensile strength, elongation at break and tensile modulus of the prepared composites were increased by 63.06%, 183.04% and 259.04%, respectively, compared with the control group. The prepared bamboo-fiber-reinforced composites have good thermal properties and water resistance, which show good application prospects in the field of industrial packaging [131].

### 8.3. Other Applications of Bamboo Fiber Reinforced Composites

**Figure 6:**
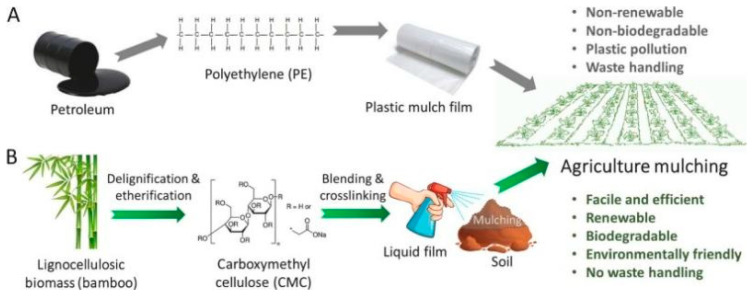
Concept of circular design of agriculture mulching using biodegradable lignocellulosic biomass-derived biopolymers to replace petroleum-derived plastics for mulch film [132].

The extensive use of plastic mulch in agriculture affects the ecological environment. A liquid film prepared from bamboo-derived cellulose (CMC) can quickly form a mulch film on the soil surface by simply spraying it on the soil (Figure 6A,B). The prepared agricultural mulch film has favorable mechanical properties, light transmission, soil water retention and biodegradability, which is promising to replace the traditional agricultural mulch film [132].

Highly customized biocomposites were fabricated by light-curing 3D printing using vinyl palm-oil-based resin as a matrix and microscale bamboo fibers (MBFs) as reinforcement. MBFs were surface grafted with methacrylic acid C=C bonds, which enhanced the dispersion and stability of the bamboo fibers in the matrix, and at the same time improved the interfacial adhesion with the matrix. The prepared bioprinted composites (5 wt% MBFs) have significantly higher mechanical strength than the pure resin, and can be degraded under mild conditions and recycled fibers, which can replace traditional 3D printing materials such as ABS plastic [133]. This study provides a direction for the green development of 3D printing.

Although the application of BFRPC is promising and significant for sustainable development, the preparation process of BFRPC is complicated, and its performance is affected by many factors, such as the content and type of bamboo fibers, the interfacial compatibility with polymers, and the molding technology. The current research on BFRPC is still insufficient, and a large number of experimental studies are still needed to explore the composite regulations between bamboo fibers and polymers, so as to make its preparation theory and technology more mature.

## 9. Conclusions

Natural bamboo fiber has many advantages such as a large aspect ratio, high specific strength, large surface area, low density, and being inexpensive, renewable and biodegradable. The development and preparation of environmentally friendly and biodegradable BFRPC using bamboo fibers as reinforcement and biodegradable polymers as substrate has become a hot research topic. Bamboo-fiber-reinforced composites have a promising prospect due to their light weight and high strength, low energy consumption, corrosion resistance and environmental friendliness. Composites produced using different polymer matrices and bamboo fibers show good mechanical properties and have obvious advantages over glass fibers in terms of cost, environmental protection and density. However, the surface of bamboo fiber is relatively rough and polar, resulting in weak interfacial bonding of composites. Modifications can be made to the surface of bamboo fibers by alkali treatment, plasma treatment, coupling agent, alkali treatment, and particle filling to enhance the bonding between bamboo fibers and the matrix. The mechanical properties of modified BFRPC, such as bending strength, impact strength and elastic modulus, have been significantly improved, which shows broad application prospects in the fields of automobile interior, product packaging, degradable disposable tableware, agricultural plastic film, construction and so on, and can replace traditional plastics to promote low-carbon life and social sustainable development. However, the production technology of BFRPC is not mature enough, and it is necessary to investigate more advanced technology to make BFRPC large-scale, industrialized and automated, to promote the wide application of BFRPC in more fields, and achieve “replacing plastics with bamboo” to promote the green development of the economy.

## Figures and Tables

**Table 1:** Mechanical properties of natural-fiber-reinforced composites.

Fibers	Matrix	Fiber Addition (wt%)	Tensile Strength (MPa)	Flexural Strength (MPa)	Impact Strength (kJ/m^2^)	Elongation at Break (%)	Reference
Bamboo	PP	40	61.85	66.25	70.75	—	[20]
Bagasse	PP	10-40	15.1	57	—	—	[21]
Jute	Melamine	16	44	112	13	—	[22]
Vetiver/Banana	Vinylester resin	—	47	86	100	4.29	[23]
Sisal	PLA	40	200.44	216.77	54.47	—	[24]
Hemp	Polyurethane	40	27.23	22.14	—	8.85	[25]
Jute/Flax	PLA	40	61.89	—	—	1.9	[26]
kenaf/Pineapple	PP	30	22.89	53.77	38.25	—	[27]
Oil palm	Plastic ABS	3	31	28	8.0	—	[28]

## Data Availability

No new data were created or analyzed in this study. Data sharing is not applicable to this article.
